# Polyphenols by Generating H_2_O_2_, Affect Cell Redox Signaling, Inhibit PTPs and Activate Nrf2 Axis for Adaptation and Cell Surviving: In Vitro, In Vivo and Human Health

**DOI:** 10.3390/antiox9090797

**Published:** 2020-08-27

**Authors:** Joseph Kanner

**Affiliations:** 1 Department of Food Science, ARO Volcani Center, Bet Dagan POB 6, Israel; jokanner@gmail.com; Tel.: +972-50-622-0761; 2 Institute of Biochemistry, Food Science and Nutrition, Faculty of Agriculture Food and Environment, The Hebrew University of Jerusalem, Rehovot POB12, Israel

**Keywords:** polyphenols, H_2_O_2_, cell signaling, PTPs, Nrf2, hormesis, Eustress, Distress, redox homeostasis

## Abstract

Human health benefits from different polyphenols molecules consumption in the diet, derived mainly by their common activities in the gastrointestinal tract and at the level of blood micro-capillary. In the stomach, intestine and colon, polyphenols act as reducing agents preventing lipid peroxidation, generation and absorption of AGEs/ALEs (advanced glycation end products/advanced lipid oxidation end products) and postprandial oxidative stress. The low absorption of polyphenols in blood does not support their activity as antioxidants and their mechanism of activity is not fully understood. The results are from in vitro, animal and human studies, detected by relevant oxidative stress markers. The review carries evidences that polyphenols, by generating H_2_O_2_ at nM concentration, exogenous to cells and organs, act as activators of signaling factors increasing cell Eustress. When polyphenols attain high concentration in the blood system, they generate H_2_O_2_ at µM concentration, acting as cytotoxic agents and Distress. Pre-treatment of cells or organisms with polyphenols, by generating H_2_O_2_ at low levels, inhibits cellular PTPs (protein tyrosine phosphatases), inducing cell signaling through transcription of the Nrf2 (nuclear factor erythroid 2-related factor 2) axis of adaptation and protection to oxidation stress. Polyphenols ingestion at the right amount and time during the meal acts synergistically at the level of the gastrointestinal tract (GIT) and blood system, for keeping the redox homeostasis in our organism and better balancing human health.

## 1. Introduction

Epidemiological, clinical and animal studies have supported a role of polyphenols in the prevention of chronic diseases, such as cardiovascular, diabetes, neurodegenerative and cancer. Paradoxically, polyphenols are barely absorbed in the gastrointestinal tract (GIT) and they undergo extensive metabolism in the GIT lumen, enterocyte and liver [1,2]. The low absorption and concentration of these compounds and metabolites in blood and peripheral tissues does not support their activity as competitive reducing antioxidants [3], and their mechanism of activity in humans is not yet fully understood. Polyphenols are plant secondary metabolites exhibiting central functions in plant protection against various biotic and abiotic stresses by their potential to act as reducing agents, activate signaling factors and interact with cytotoxic agents. In plants, they have antimicrobial, antiviral, antifungal, anti-insects, anti-herbivores, wound-healing, drought and UV protection properties [4]. Polyphenols are naturally occurring compounds present in fruits, vegetables, spices and beverages (tea, coffee, red wine), and are the most abundant reducing compounds ingested in the human diet, and the total intake was re-estimated to be about 1000 mg/day [1,2]. Dietary intake of polyphenols was also estimated in a Spanish population by a PREDIMED study and was found to be 820 mg/d, of which 443 mg/d were flavonoids, 304 mg/d were phenolic acids and 73 mg/d belonged to other polyphenols groups [5]. Polyphenols can be classified into several classes, that include phenyl-propanoides, and their number in plants is more than 10,000. Phenolic compounds are those that have at least one benzoic ring with one or more hydroxyl groups. The amino acid phenylalanine is the precursor to all the polyphenols which are biosynthesized by specific enzymes to non-flavonoids, such as phenolic acids (gallic acid), stilbenes (resveratrol), lignans (sesamin) and ellagic acids (ellagic acid), and flavonoids such as chalcones (phloretine), flavanones (naringenin), flavones (apigenin), dehydroflavonols (dehydroquercetin), flavonols (quercetin) and anthocyanins (cyanidin). A part of the flavonoids are condensed to tannins such as procyanidins and proanthocyanidins, which could be dimers, small oligomers or polymers. Hydrolyzable-tannins (gallotannins or ellagitannins) are polymers readily hydrolyzed into their components: glucose, flavonoid and carboxylic acid [2].

There are several controversies as to how polyphenols activate signaling factors. These include:

(1) Polyphenols are inhibitors of NADPH oxidases (NOXs) [6] (“active” only after auto-oxidation to quinones [7,8]).
(2) Polyphenols inhibit enzymes and affect receptors by interaction with the protein molecule [9,10].
(3) Polyphenols are inducers of intracellular generation of reactive oxygen species (ROS) [11,12].
(4) Exogenous to cells polyphenols by generating H_2_O_2_ at 0.1–5 µM activate the nuclear factor erythroid 2-related factor 2 (Nrf2) signaling factor [13].
(5) Polyphenols, by generating quinones and by interaction with SH-Keap1 protein, activate Nrf2 [14].

We suggest that polyphenols, by generating H_2_O_2_ in the blood system, at the endothelial cell membranous exogenous area, act as activators of signaling factors, increasing cell adaptation and survival. The polyphenols’ actions in the blood system are dependent not only on the generation of H_2_O_2_ but also on its concentration formed. Exogenous generated H_2_O_2_ enters cells through aquaporin [15], the protein channels generally associated with water transport. Because the low bioavailability of nutritional polyphenols and metabolized compounds in the human blood system are mostly around 0.1–2 µM [1,2], they act at the level of the cell membrane by generating H_2_O_2_, penetrating in cells by 0.01–0.1 µM to improve adaptation and survival of the organism.

## 2. Polyphenols as Reducing Agents and Pro-Oxidants

In general, polyphenols, as reducing agents and pro-oxidants, at the level of the GIT and blood system, act synergistically for keeping the redox homeostasis in our organism and better affecting human health. After ingestion, polyphenols affect human health by their action in the stomach, GIT (gastrointestinal tract) and in the blood system. In the GIT, they act as (a) reducing agents, preventing lipid peroxidation and generation of AGEs/ALEs (advanced glycation and lipid oxidation end-products) [16], and (b) compounds affecting the activity of GIT enzymes, configuration of functional and non-functional proteins and gut microbiota spectrum, by hydrogen-bonding and hydrophobic forces [17]. In the blood system, and especially in the blood micro-capillary area, in paradox, they act at very low concentration as (a) generators of H_2_O_2_, reacting in different organs (cardiovascular system, liver, pancreas, lung, kidney and blood–brain barrier cells) as cell signaling [13], and (b) cytotoxic agents [18,19]. When polyphenols in the blood system attain high concentration ~≥5 µM, they generate relatively high concentrations of H_2_O_2_ and other derivatives, acting to induce oxidative stress [20].

Why are polyphenols such good reducing agents with the potential to act as pro-oxidants? These broad effects are connected with the specific structure and electronic configuration of the hydroxyl oxygen bound to the benzene ring molecule. The electrons on the valence orbitals of an oxygen atom, before bonding to one of the benzene carbons, undergo sp^3^ hybridization, which forms two orbitals with two pairs of non-bonding electrons and another two orbitals with un-paired electrons which form two ơ bonds, one with hydrogen and the other with the benzene carbon ring. There is considerable evidence that intra-molecular and inter-molecular H-bonding to the pair’s non-bonding electrons of oxygen has a pronounced effect, which increases its redox activity. Active polyphenols owe their activity to a combination of electronic and steric effects which lower the bond dissociation enthalpy (BDE) of the O-H bond, which increases its reaction with peroxyl or alkoxyl radicals [21,22,23,24]. Polyphenols have high free radical scavenging activity but also act in the presence of oxygen and metal ions to generate O_2_**^•−^** and H_2_O_2_ by the following reactions:

Ph-OH + electrophiles/M^+n^/R^•^ → Ph-O^•^ + reduced compounds (1)

Ph-O^•^ + O_2_ → Ph = O + O_2_^•−^ (2)

Ph-O^•^ + O_2_^•−^ → Ph = O + H_2_O_2_ (3)

Ph-OH + Ph=O → 2 Ph-O^•^ (4)

where M^+n^ = oxidized metal ion; R^•^ = free radical.

The resulting phenoxyl radical must be sufficiently stable or have a redox potential which does not initiate a new chain reaction. One of the features that stabilizes the phenoxyl radical is the aromatic structure of the benzene ring that allows formation of aroxyl radicals by resonance. The reaction between a phenoxyl with a peroxyl radical is 300-fold less rapid than between a phenoxyl radical with an aroxyl, generating a phenoxyl and aroxyl radical [25]. This reaction is easily adjusted when polyphenols are in a very broad mixture, such as in natural plant material generating a synergistic redox effect, by the reaction (5). Even at low concentration, a very active reducing polyphenol, in mixture with other less active polyphenol, will present a higher redox effect.

PhO^•^ + ArOH → PhOH+ ArO^•^ (5)

However, kinetic aspects should be considered for the reducing effects of polyphenols because redox activity in biological systems are very much affected by metal or enzyme catalysis, membrane structure, molecule solubility and polarity, pH, water-activity, bioavailability and metabolism. For these reasons, determination of reducing activity in a food or nutrient system should be not only a test but a thorough full study.

## 3. Polyphenol Auto-Oxidation without the Involvment of Metal Ions

**Figure 1:**
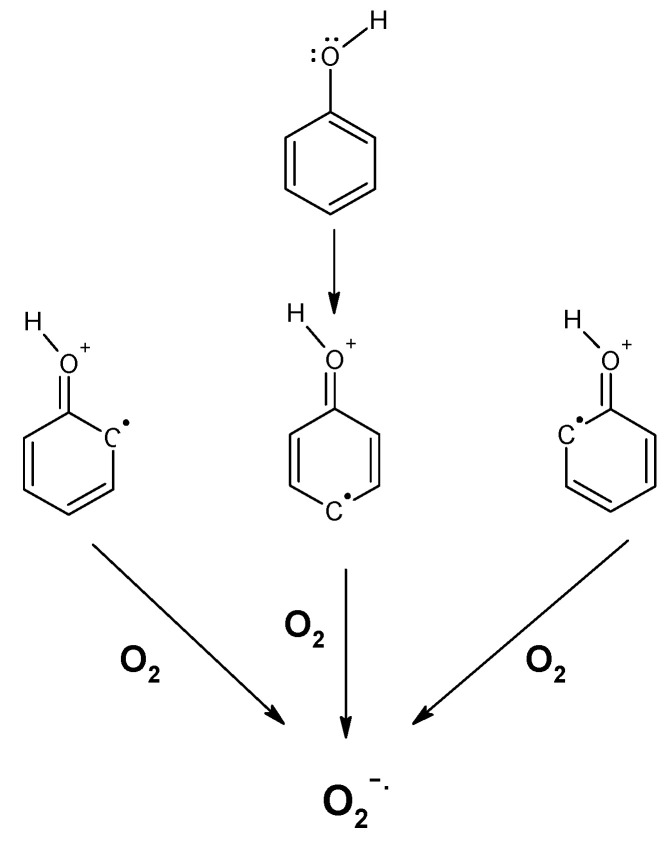
Polyphenols (hydroquinone) are present in tautomeric and three resonance states.

The intra- or inter-molecular H-bonding to the O-H groups around the polyphenols also increases its tautomeric effects, generating resonance to four forms of the phenol molecule [21,22,23]: one, the regular structure, and the other three, hydroquinone cation radicals (HQ+**^.^**), containing an un-paired electron on ortho-, meta- or para-positions of the benzene ring (see reactions (1)–(4) and Figure 1).

The new structures act to lower the bond dissociation enthalpy (BDE) of the O-H bond, increasing the reducing capability of the molecule. There is considerable evidence that intramolecular H-bonding, such as in the catechol, or better, in the galloyl form of the ring system, has a pronounced effect on the reducing activity.

**Figure 2:**
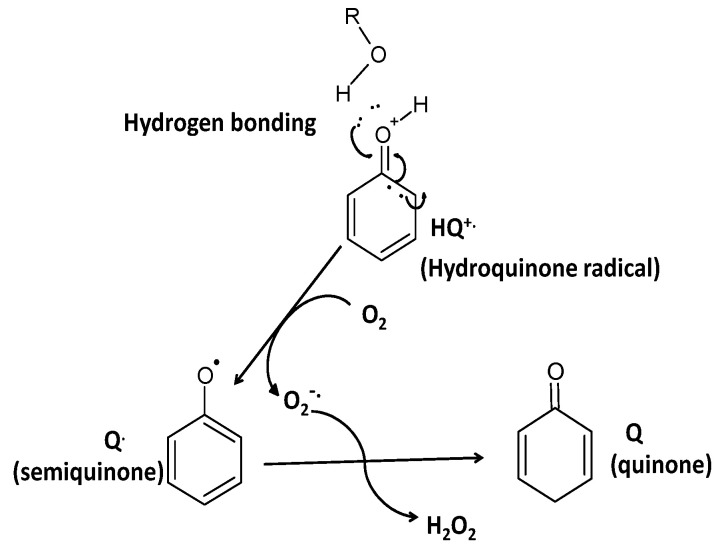
The effects of hydrogen bonds and conversion to hydroquinone cation radical and subsequently, production in the presence of oxygen the hydrogen peroxide.

**Figure 3:**
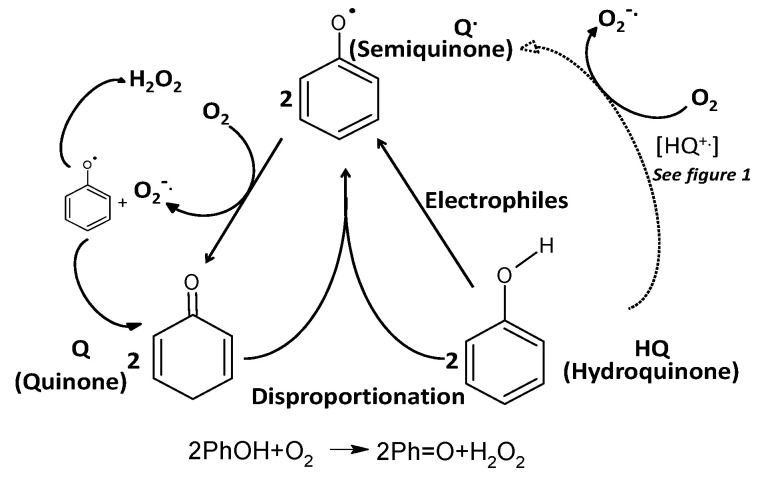
The auto-oxidation and disproportionation of polyphenols to two semiquinone radicals, generating H_2_O_2._

The electronic configuration of the hydroquinone cation radical containing an unpaired electron around the benzene ring opens the possibility of direct interaction with ^3^O_2_ (triplet) oxygen, without the need of a metal ion as a bridge for electrons transfer. The electronic configuration of oxygen is very unique, containing at the 2π anti-bonding orbitals of two un-paired electrons forming a triplet state, paramagnetic and free biradicals. The ^3^O_2_ could interact freely with free electrons, transition metals, free radicals and triplet molecules, however not with molecules at the singlet state, such as polyphenols or most of the other 99.9% of the known molecules, because of the electron spin forbidden effect [26,27]. The tautomeric effect generating the hydroquinone radicals allows for direct interaction with ^3^O_2_, forming super-oxide anion radicals, perhydroxyl radicals (at pH < 4.5), H_2_O_2_, semi-quinones and quinones. The generation of quinones in the presence of reduced polyphenols (but also other reducing agents) and ^3^O_2_ initiates the pathway for auto-oxidation of polyphenols (Figure 2 and Figure 3).

One possible route of these electrophiles in the organism, at very low concentration, is activation of transcription factors. At relatively high concentrations of polyphenols, the pro-oxidative route is cytotoxic, capable of modifying proteins and DNA. The geometric isomers of hydroquinones for activation to electrophiles are important, because only the ortho- and para-hydroquinones, but not meta-forms, are active to generate electrophiles [28]. The capability of polyphenols to act as chelating agents allows them to also affect the allocation of transition metals such as iron, copper, zinc and others in the cells and act together to enhance oxygen activation to free radicals. Chelating agents such diethyl-dithiocarbamate were found by allocation of copper ions in liver of mice to enhance the polyphenol–copper redox-reaction and the cytotoxic effects of the polyphenol on liver of mice [20]. Polyphenols alone or by interactions with polyphenol’s derivatives allowed them to act at the same time as reducing agents and generators of active oxygen species. It is hypothesized that at low concentrations of ~0.1–1 µM in the blood system, the polyphenol antioxidant effect is not significant, but the generation of H_2_O_2_ at the exogenous level of the endothelium, after diffusion trough aquaporins, affects endothelial cell signaling factors and has an important effect on transferring cell signaling, affecting the organ redox system.

## 4. The Pro-Oxidant Action of Polyphenol in the Cardiovascular System and Organs

The ultrastructure of terminal mammalian arterioles is composed of endothelial cells which have the same height and reach at the thinnest area, a diameter of ~0.15 micron [29]. The arteriole ultrastructure permitted the blood system to be well connected with our organs for a perfect transfer of nutritional elements and exchange of small or gas molecules, especially H_2_O, O_2_, CO_2_, H_2_S and NO. This ultrastructure also permitted the polyphenols to be in high interaction with the endothelial cell membranes, most probably by the hydrophobicity or hydrogen bonding between polyphenol hydroxyls and protein or phospholipid amine groups.

Due to the poor absorption and extensive metabolism in the enterocyte, polyphenols undergo extensive metabolic transformation, but still retain significant redox capabilities and could generate H_2_O_2_ [30,31,32]. The bioavailability of a specific polyphenol molecule from the GIT into the blood system could attain a concentration of ~100 nM, but in ensemble with other polyphenols, they could reach a higher concentration of ~1 µM and even more. It is most likely that polyphenols may act in vivo via the pro-oxidative effects, following reactions which generate H_2_O_2_, semiquinones and quinones [13]. At the extracellular endothelial cell membranes area, SOD_3_ (the extracellular enzyme) transforms O_2_^•−^ to more H_2_O_2_. Hydrogen peroxide, generated at the extracellular area, will diffuse across membranes through aquaporins, known as peroxiporins [33], into endothelial cells, affecting redox cellular responses via activation of signaling factors. H_2_O_2_ is a well-accepted second messenger [13,34,35]. Thus, for H_2_O_2_ to play a direct role in signaling, its target(s) must be localized near its site of production, especially because of the high cellular enzymatic activity of peroxidases. Quinones and semi-quinones are generated during auto-oxidation of polyphenols, but because of high affinity to blood particles and membrane proteins, the diffusion across membranes seems to be much lower than H_2_O_2_ [8,36]. After diffusion inside the cells and near the membrane, a very low concentration of ~10 nM H_2_O_2_ will affect cell proliferation and angiogenesis. Hydrogen peroxide at concentrations of 10–100 nM will affect adaptation to stress responses, but at higher concentrations of 1 µM H_2_O_2_ and more, it will induce inflammation and cell death [13,37,38]. Those processes were demonstrated in vitro, and in part in vivo, to be affected by various polyphenols.

## 5. Cell Proliferation, Inhibition and Progression by Polyphenols

The possible beneficial health effects of diets containing polyphenols have led to an enormous scientific interest in those compounds. Eberhardt et al. reported inhibition of tumor cell proliferation in vitro by polyphenols, extracted from apples, and published those results in the *Nature Journal* [39]. The suggestion that these compounds inhibit proliferation of tumor cells was not examined critically. We found that those effects were developed by interaction of polyphenols with the cell culture media, in vitro, generating H_2_O_2_. These effects were inhibited by catalase and myoglobin [19,38]. Several researchers demonstrated that polyphenols at very low concentration, in cell culture, generate low concentrations of H_2_O_2_, which increases cell proliferation, wound repair and survival; however, at high concentration due to high generation of H_2_O_2_, they inhibit proliferation, angiogenesis, wound repair and decrease cell survival [12,19,40,41]. Reaction of redox-active compounds with cell culture media, in vitro, to produce reactive oxygen species generating artifact results, are not unique to polyphenols, and they were observed with other reducing compounds and reviewed by Halliwell [42]. One should consider that after ingestion, dietary polyphenols and metabolites, still with reducing power, could interact with the cell membranous area of the gastrointestinal tract or after absorption with the blood endothelial system, generating H_2_O_2_ and other reactive oxygen species, mostly at low concentration, affecting significant physiological changes and cell signaling. Many studies considered the effects of polyphenols in different systems without using specific inhibitors for critically understanding the mechanism and the real active compound responsible for the results accepted, [40,41,43,44,45]. Several other studies, however, by introducing specific inhibitors, such as catalase, myoglobin, Peg-catalase, SOD (superoxide dismutase), Peg-SOD and MnTMPyP (manganese (III) 5,10,15,20-tetra(4-pyridylporphyrine)) [12,19,46,47,48,49,50,51,52], identified H_2_O_2_, the active specific by-product, generated by auto-oxidation of polyphenols, affecting the physiological changes obtained. We demonstrated that catalase decreased H_2_O_2_ levels generated by glucose-oxidase, but it could not cause the same effects when H_2_O_2_ was generated by polyphenols [38]. This discrepancy is of note because glucose-oxidase generated H_2_O_2_ in the aqueous medium and polyphenols generate H_2_O_2_ mostly by site, specifically on the cell membrane. To inhibit such H_2_O_2_ flux, myoglobin, a cationic protein, was found to interact with the negatively charged membranes, which makes it a more efficient H_2_O_2_ decomposer than catalase, which has a low affinity for membranes because of its negative charge [38,53].

## 6. Adaptation, Protection and Cell Survival; In Vitro

It seems that most polyphenols, by generation of H_2_O_2_ [19,41] at low concentration by preconditioning in cell cultures, protect cells from high H_2_O_2_-induced cell cytotoxicity (Table 1). Preconditioning of cells with H_2_O_2_ (10 µM) was found to protect the same cells from the subsequent addition of 6 mM of H_2_O_2_, which, without preconditioning, induced cytotoxicity [54]. H_2_O_2_-preconditioning of cells was found to modulate phase-II-enzymes through PI3K/Akt kinase (phosphatidylinositol-3 kinase/protein kinase B), MAPK (mitogen-activated protein kinase) and JNK (c-Jun N-terminal kinase) activation [55,56]. This activation of protein kinases prevents cells from cytotoxicity affected by many toxic factors. H_2_O_2_ at concentrations of 1–5 µM generated from t-BHQ (tert-butylhydroquinone), resveratrol and curcumin, activates Nrf2 and the phase-II-enzymes in astrocytes, but were prevented by met-Mb (met-myoglobin) and glutathione [13], two compounds which do not diffuse through the cell membrane. Shifting of the redox environment by a pro-oxidative effect of resveratrol generated protection of cells against stress [57]. Many other polyphenols were found in vitro to prevent cell cytotoxicity, most probably via generation of H_2_O_2_ at low concentrations. Baicalein protects cardiomyocytes or neuroblastoma cells from hypoxia reoxygenation and H_2_O_2_-induced cytotoxicity respectively, by generation of H_2_O_2_, demonstrated by inhibition with SOD and catalase [45,58]. The pretreatment with baicalein up to 10 µM activates protection, most probably because of the generation of low concentrations of H_2_O_2_, while pretreatment with 100 µM was ineffective.

Neuroprotective effects of the citrus polyphenols, hesperidin and hesperitin, were found to protect against H_2_O_2_-induced cytotoxicity in PC12 cells without determining the possible pro-oxidative effect [61]. Human retinal pigment epithelial-19 cells were found to be protected from H_2_O_2_ or t-BOOH (tert-butyl-hydroperoxide) by preconditioning of the cells with anthocyanins or the flavonoid Eriodictol, respectively [64,66]. Preconditioning with blueberry polyphenols was found to protect neuroblastoma cells from H_2_O_2_-induced cell injury by activation of PI3K/Akt, MAPK, p38 (p-38 mitogen-activated protein kinase), and the activity loss of ERK1/2 (extracellular signal-regulated kinase 1/2) and MEK1/2 (mitogen-activated extracellular kinase 1/2) [65]. Preconditioning with quercetin causes Nrf2 nuclear translocation, increases glutathione levels and prevents neuronal death against H_2_O_2_ cell injury [63]. Resveratrol protects cardiomyocytes, H9c2 cells and astrocytes from H_2_O_2_-induced cell injury and apoptosis by activating the expression of SirT1 (sirtuin 1 deacetylase), FoxO1 (forkhead box O1) and HO1 (heme-oxidase 1), respectively [59,60,67]. Hydroxytyrosol, the main polyphenol in olive oil and leaves, inhibits H_2_O_2_-induced cell injury in vascular endothelial cells by activation of kinases and expression of Nrf2, which induces HO1 and upregulates catalase expression through the AMPK/FoxO3a (5-AMP-activated kinase/forkhead box O3) pathway [38,60]. The last studies were critically examined by Schaffer and Halliwell [68], who found that all the polyphenol effects were generated by H_2_O_2._ Elbling et al. [12] elegantly demonstrated that human keratinocytes were protected from cytotoxicity of high concentrations of polyphenols or H_2_O_2_ ~ 50–100 µM by preconditioning of the cells with low concentrations of polyphenols or H_2_O_2_ ~ 1–5 µM. This protection was prevented by catalase, which demonstrated that the polyphenol effect arises mainly due to generation of H_2_O_2_.

## 7. Adaptation, Protection and Cell Survival; In Vivo

Polyphenols, due to generation of H_2_O_2_, act for adaptation and protection not only in in vitro cell culture but also in-situ and in vivo, with animals and humans. Table 2 summarizes, in part, the relevant oxidative stress markers for evaluation of cell signaling and transcription factors affecting genes inducing synthesis of proteins, enzymes or cytokines. An increase in eNOS (endothial-nitric oxide synthase) expressions in the aorta was observed in in vivo studies by intake of wine polyphenols in rats [69]. Treatment of mice with apple polyphenols or one of the main tea polyphenols, epigallocatechin gallate (EGCG), before exposing the animals to CCl_4_, prevented liver cytotoxicity [70,71].

Other polyphenols such as baicalein, hibiscus polyphenols, grape seed polyphenols and the marine polyphenol, dieckol, protect liver cytotoxicity by CCl_4_ in mice [73,74,75,76,77,78,79]. Lipopolysaccharide (LPS), the endotoxin produced by all Gram-negative bacteria, induces inflammation by activating immune cells to produce inflammatory cytokines and macrophages to produce inflammatory mediators [80]. Preconditions with polyphenols in several in vitro and in vivo studies prevent generation of pro-inflammatory cytokines by LPS in microglial cells [43] and epithelial cells [44], and in mice: neuro-damage effects [79], hepatic injury [76], paw edema [78], anti-inflammatory capacity [81] and sepsis [77] (Table 2). The data reviewed above help to summarize markers for oxidative stress in in vitro and in vivo studies (Table 1 and Table 2).

## 8. Polyphenols and Cardiovascular System, Ex Vivo

Epidemiological studies have indicated that regular intake of polyphenol-rich food and beverages, such as fruits, vegetables, red wine, tea or cocoa, is associated with a reduced risk of cardiovascular diseases [82,83]. The dietary intake of polyphenols is highly variable, and all the foods contain many classes of polyphenols. Due to catabolic and metabolic reactions of the parent compounds, the absorbed constituents in the blood vessels, at a low micromolar concentration, retain, in part, the reducing potential and the possible interaction and synergism between polyphenols (reaction (5)) to generate H_2_O_2_. As is known, like H_2_O_2_ [84,85,86,87], polyphenols, through intercellular generation of H_2_O_2_ and intracellular increase of H_2_O_2_ [11,13,48,88], affect endothelial formation of NO and endothelial-dependent hyperpolarization (EDH), both of which induce vaso-relaxation. In the blood system, the endothelial cells are most affected by the action of polyphenols due to higher interaction with the membranous proteins. Several studies demonstrated that grape/wine polyphenols, due to a pro-oxidant effect, generate ROS into endothelial cells, which affect redox-cysteine-sensitive upregulation of eNOS by activation of PI3-kinase/Akt, p38, MAPK and JNK, and inactivation of FoxO1 and FoxO3a [11,50]. These effects are induced through activation of the PI3-kinase/Akt/eNOS pathway, which generates NO [11], inducing vaso-relaxation. All these effects were prevented by PEG-catalase and MnTMPyP [11], which interact with the cell membranes and better decompose the H_2_O_2_ generated by the membranous-interacted polyphenols [13].

## 9. Several Other Effects of Polyphenols/H_2_O_2_ in Animal and Human Organisms

Vauzour et al. [10] found that hesperetin in very low concentration activates PI3K/Akt and ERK1/2 in neurons via inhibition of the phosphatase PP2A (protein phosphatase 2A). The authors suggest that the inhibition was most probably by interactions with the active site of the enzyme. One could suggest, however, that this effect was derived due to generation of H_2_O_2_ by hesperetin [89]. It is well known that low concentrations of cell exogenous H_2_O_2_ (50–100 nM) inhibits PP2A and other PTPs (protein tyrosine phosphatases) [90,91], thereby increasing the level of protein phosphorylation [91,92,93]. It seems that nano-molar concentration of caffeic acid/H_2_O_2_, which partially inhibits PP2A, increases phosphorylation and nuclear Nrf2, and decreases nuclear p65 (protein 65), and in this way, prevents deregulations of the cells by high glucose. Nano-molar concentration of caffeic-acid attenuates glucose-induced endothelial cell dysfunction by affecting NF-kB and Nrf2 pathways [88]. Nrf2-mediated inhibition of the inflammatory cytokine gene expression is ARE-independent. Nrf2 specifically inhibits the inflammation-induced transcription mediated by NF-kB. This notion coincides with the fact that Nrf2 also binds to the Interleikin-6 and Interleukin-1b (IL-6 and IL-1b) genes’ loci and inhibits their transcription [94]. In general, at the same time, Nrf2 upregulates expression of genes coding antioxidant proteins and downregulates target genes that encode inflammatory cytokines, and in this way, eliminates ROS and subsequently contributes to the anti-inflammation process [94]. Epigallocathechin-3-galate (EGCG) at sub-micromolar concentration suppresses hepatic gluconeogenesis through H_2_O_2_ activation (which was prevented by PEG-catalase and MnTMPyP) of AMPK mediated by CaMKK (Ca/calmodulin-dependent protein kinase kinase) [47]. Very similar to H_2_O_2_, polyphenols activate formation of NO through Ca/Calmodulin [95,96], activate estrogen receptor [97,98], CaMKK, AMPK and SirT1 [96,99].

Many evidences support a potential beneficial action of polyphenols consumption on cardiovascular health [100] and type 2 diabetes mellitus [101,102]. In mice fed a high-fat diet, Daveri et al. [103] have shown that polyphenols modulate inflammation and alter redox signaling, improving insulin resistance. Several studies in vivo on tea polyphenols, and especially EGCG, via dampening of PTP1B (protein tyrosine phosphatase 1B) and other PTPs acting as key regulators of tyrosine phosphorylation-dependent signaling accelerate glucose uptake and evoke the IRS-1/Akt/GLUT2 signaling pathway in HepG2 cells and mice liver [104]. By inhibition of PTP1B, EGCG stimulates nuclear translocation of Nrf2 after provoking the PI3K/Atk signaling pathway, and thus modulates the expressions of antioxidant enzymes such as HO-1 and NQO1 [104], most probably by activation of Nrf2 transcription. Furthermore, EGCG supplemented to mice significantly ameliorated high-fat high-fructose diet (HFFD)-triggered insulin resistance and postprandial oxidative stress, cognitive defects by upregulating the IRS-1 (insulin-receptor substrate 1)/Akt, Keap/Nrf2 and ERK/BDNF/CREB (brain-derived neurotrophic factor/c-AMP-response element binding protein) transcription pathways [104,105]. In mice, EGCG also ameliorates the metabolic syndrome derived from HFFD, by increasing brown adipose tissue (BAT) energy expenditure and preventing adipocyte hypertrophy and fat accumulation [105,106]. BAT is a major regulator of thermogenesis in mammals. A high-fat diet (HFD) was found to promote the growth of flavonoid-metabolizing bacteria, which in turn decrease the amounts of bioavailable flavonoids which are important to ameliorate post-dieting obesity. Interestingly, weight-adjusted energy expenditure was markedly reduced in weight-cycling mice, but was normalized upon flavonoid administration [107,108,109]. The research shows that after two weeks of administration, apigenin and naringenin (and not the catabolized flavonoid compounds) significantly elevated the thermo-genic factor uncoupling protein-1 (UCP1) transcript levels in BAT of mice fed the HFD. Since other flavonoids (quercetin, hesperetin, epicatechin apigenin, blackcurrant anthocyanins, theaflavins, chrysin) have been previously associated with the induction of the major UCP1 in BAT [110,111,112], it seems that this is an important pathway by which flavonoids may affect overweight. However, one should emphasize that the activation of UCP1 is also induced by H_2_O_2_, generated by auto-oxidation of phenols [113,114]. Interesting results were published on the possible therapeutic potential of aspirin beyond its ability to inhibit cyclooxygenase pathways. The researchers found that aspirin and salicylic acid are partially metabolized to di-hydroxy-benzoic acid (polyphenol), generating H_2_O_2_, which acts as an inducer of Sirt1 and other downstream targets of Sirt1, PGC-1α and AMPK [113,114].

Circulating levels of glucose and free fatty acids are increased in patients with type 2 diabetes mellitus (T2DM) and metabolic syndrome [88,101,102,103,115,116,117]. These effectors activate the generation of ROS, activation of NF-kB (nuclear factor kappa B) and the pro-inflammatory pathway through phosphatase and NADPH oxidases (NOX1 or NOX4) [96,118,119]. Polyphenols seem to ameliorate this deleterious pathway by generating low concentration of H_2_O_2_ in arterioles, which interacts exogenously with endothelial cells, penetrates into cells and inhibits phosphatases [118], increasing phosphorylation of several anti-inflammatory signaling factors and especially the Nrf2 target genes. These activities are not relevant to the blood system alone, but because the blood system attains all the organs, it seems to beneficially affect all of them. It seems that the involvement of polyphenols as a pro-drug generating low concentration of H_2_O_2_ acts beneficially in several more systems.

## 10. Polyphenols and Brain Function

Polyphenols were found to ameliorate age-related cognitive decline and neurodegenerative diseases. The beneficial effects of polyphenols on brain function seem to act by modulating signaling pathways, promoting cerebrovascular blood flow, controlling synaptic plasticity, reducing neuro-inflammation, stimulating new nerve cell growth and attenuating extracellular accumulation of pathological proteins. There are several studies on bioavailability of polyphenols in brain tissues founding some transfer, but further work is necessary to confirm that polyphenols can diffuse in the brain and directly modulate brain function [120]. Hesperitin was found to affect Akt and ERK1/2 activation status in cortical neurons [10]. In mice, hesperidin was found to attenuate learning and memory deficiency in APP/PSI mice (β-amyloid precursor protein/presenilin1) through activation of Akt/Nrf2 signaling and inhibition through receptors of advanced glycation end-product (RAGE), which activates the NOX1/H_2_O_2_/NF-kB pathway [121]. Dietary supplementation with tBHQ (tert-butylhydroquinone), an Nrf2 activator, confers neuroprotection against apoptosis in amyloid β-injected rats [122]. tBHQ was found to confer neuroprotection in vivo in several more studies [123,124,125]. Curcumin provides neuroprotection in in vivo models of traumatic brain injury and cerebral ischemia-reperfusion via p-Akt and p-mTOR (mammalian target of rapamycin) and the Nrf2-ARE (antioxidant response element) signaling pathways [126,127]. Resveratrol was also found to confer neuroprotection in mice against aging-related deficits through an ERK1/2-dependent mechanism [128]. All these polyphenols are different in molecular configuration from different classes and molecular size but all generate H_2_O_2_ by auto-oxidation [13]. The protective effects it seems were mediated by an indirect mechanism, affected by an exogenous low concentration of H_2_O_2_ flow, generated at the level of the blood–brain barrier (BBB) cells. Polyphenols such as tBHQ, curcumin and resveratrol activate the Nrf2 pathway in astrocytes by exogenous H_2_O_2_ [13]. The importance of exogenous H_2_O_2_ generation delivering redox signaling for healing of axons was recently published [129,130]. Hervera et al. [129] identified a new physiological role for H_2_O_2_ in the brain in which it acts as trans-cellular signaling, established by exosome-mediated NOX transfer as a mechanism for this pathway. The exosome is generated from macrophages, recruited and attracted to the localized tissue injury, which produces H_2_O_2_, and helps to transfer the effect at distant. The generation of H_2_O_2_ at low concentration, from the exosome, causes oxidation-induced inactivation of PTEN (phosphatase and tensin homolog), which enables activation of the PI3K/p-Akt signaling pathway, and leads to adaptation and enhanced survival for cells and beneficial effects for the brain. The experiment in mice [121] by which polyphenols attenuate learning and memory through activation of Akt/Nrf2 signaling and inhibition of the RAGE/NF-kB pathway, integrates two main factors affected by polyphenols, one, the action mainly induced in GIT, preventing generation of cytotoxic aldehydes, AGEs/ALEs, preventing the activation of the (receptor advanced glycation factor) RAGE/NF-kB pathway, and the other in the blood system, by which H_2_O_2_ at nM concentration activates the Akt/Nrf2 signaling. In several more experiments, polyphenols ameliorated the postprandial oxidative stress, induced by cell culture supplemented with glucose-amines or mice with the high-fat high-fructose diet (HFFD), both generating AGEs in the model system or organism [108,109]. Polyphenols act synergistically at the brain level for keeping better brain adaptation and surviving oxidative stress by decreasing lipid oxidation and generation of reactive aldehydes, AGEs/ALEs, in the GIT [16,131,132,133] and acting as a pro-drug for H_2_O_2_ in the blood system, affecting Nrf2 pathways of cell signaling and increasing generation of endogenous antioxidants.

## 11. Hormesis/Eustress and Distress by Polyphenols

Hormesis is an adaptive response characterized by biphasic dose response affected by an active compound. The hormetic actions of polyphenols in the blood system are dependent on H_2_O_2_ concentration, found to be in the range of 0.25–5 µM [37,134,135,136,137]. There are many examples of hormesis by polyphenols/H_2_O_2_. At different concentrations, they activate or inhibit the same effector [57], like HO-1 [138,139], receptor tyrosine kinase activity [92] or Nrf2 [51,58]. The activation of Nrf2 by sulforaphane was enhanced by low concentration of H_2_O_2_ or polyphenols but inhibited at high concentration of the two additives, H_2_O_2_ or polyphenols [51]. The oxidizable phenol 2,5-di-tert-butylhydroquinone (dtBHQ) is a useful tool for assessing H_2_O_2_ contribution. Both tBHQ and dtBHQ readily oxidized to produce O_2_**^•−^**, generating H_2_O_2_ either spontaneously or catalyzed by SOD3. However, only the oxidized form of tBHQ, tBQ, can act as an electrophile, since the additional tert-butyl group on dtBQ blocks its ability to react with a nucleophile [51,140]. It was found that only dtBHQ-generated H_2_O_2_ is responsible for the significant enhancement of sulforaphane-induced Nrf2/ARE-regulated gene expression [51]. These results confirm our data which show that H_2_O_2_ is the main molecule that activates astrocyte Nrf2 and not the quinones [13]. Several researchers believe that after absorption, polyphenols activate signaling factors by specific interaction between the molecules with receptors, protein kinases or transcription factors. However, because many polyphenols from different classes and molecular size activate the same factors in different cells, organs or animals, one should hypothesize a possible common pathway bringing these compounds to act in a similar fashion. Forman [14] proposed that the major mechanism of action for nutritional polyphenols is the activation of the Nrf2 signaling pathway by quinones, which react with the Keap1 of Nrf2 by a Michael addition. Experiments with endothelial cells conducted in the presence of human serum albumin prevent epicatechin activity due to strong binding to the protein [6], and even more in the presence of quinones. Because of the high reactivity of quinones with proteins, and especially with protein-SH-groups in blood [8,36], experiments with dtBHQ, and poor diffusion into cells, allow H_2_O_2_ to be the main polyphenols by-product and effector of cellular transcription responses [13] acting as an Eustress. A synergistic pro-oxidant toxic effect was generated in liver of mice by the polyphenol EGCG (45 mg/kg, intra-peritoneal) in the presence of diethyldithiocarbamate (chelator of Cu), which increases the interaction between liver-Cu ions and the polyphenol, and thus elevates generation of H_2_O_2_. This exaggerated pro-oxidative effect induces hepatic transcriptional responses, which increase pro-apoptosis and pro-inflammatory genes such as p21, iNOS (inducible nitric-oxide synthase) and COX-2 (cyclooxygenase-2), and decrease Nrf2 target genes such as NQO1 (NADPH quinone reductase), SOD1 (super oxide dismutase1), CAT1 (catalase1), Prx1 (peroxiredoxin 1), TRX1 (thioredoxin) and Gpx1 (glutathione peroxidase 1) [20]. This experiment demonstrates the possible hormetic effect of polyphenols/H_2_O_2_ in cell signaling and its possibility to also act as a Distress compound. Most recently, just during the preparation of this review, Calabrese et al. [141], by searching hormesis effects of polyphenols, confirmed our previous data and conclusions [13].

## 12. Conclusions

**Figure 4:**
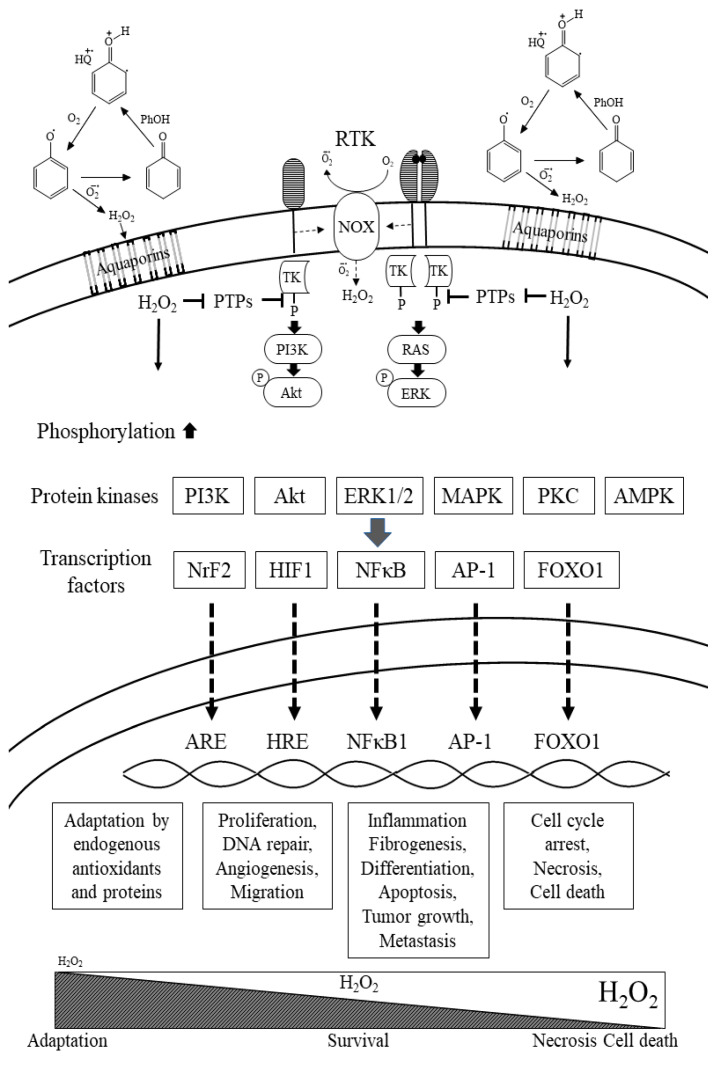
By generating H_2_O_2_, polyphenols activate cell redox signaling.

The current review tries to shed light on the mechanism by which absorbed polyphenols and reducing metabolites in the blood system affect human health, mainly by acting as a H_2_O_2_ ’’pro-drug’’. The polyphenols’ effects on the cardiovascular system and organs depend on H_2_O_2_ generation and its concentration, but also on subcellular localization, the presence of H_2_O_2_ destroying enzymes, cell-type and specific organ, intensity and time duration of the stimuli. Cells respond to multiple exogenous stimuli via various receptors, such RTKs and RAGEs, in the plasma membrane, by activating NOX-4 and SOD3, initiating H_2_O_2_, which enters the cell via aquaporins. In cells’ normal stage, the level of H_2_O_2_ generated is in the range of ~1–10 nM. However, after activation of receptors by growth factors, cytokines or nutrients, such as ALEs/AGEs, the level of H_2_O_2_ in cells increase and could be in the range of 10–100 nM, generating “Eustres” affecting DNA repair, differentiation, migration and adaptation, or in the range of >1000 nM, generating “Distress”, affecting inflammation, apoptosis, necrosis and death [142].Pre-treatment of cells or organisms with polyphenols, by acting as a pro-drug generating H_2_O_2_ at low nM concentration, inhibits cellular PTPs, inducing cell signaling trough Nrf2 pathways of adaptation and protection to various oxidation stress factors, keeping Eustress. Most human health benefits from different polyphenols molecules’ consumption in diet are derived by their common activities in the stomach and GIT, in the cardiovascular system and at the level of the blood micro-capillary. In the stomach, intestine and colon, polyphenols act as reducing agents, preventing lipid peroxidation and generation of AGEs/ALEs, preventing postprandial oxidative stress [16,132,143]. They also act as compounds affecting the GIT enzyme activity, enterocyte transcription factors, nutrient absorption and gut microbiota spectrum [16,17,132,133,144]. In paradox, in the blood system at very low concentration, polyphenols act as generators of H_2_O_2_, affecting cell signaling, adaptation and survival. When polyphenols attain high concentration in the blood system, they generate relatively high concentration of H_2_O_2_ and possible other derivatives, acting as cytotoxic agents inducing Distress (Figure 4).

A variety of cell surface receptors induce protein phosphorylation. Receptors for peptide growth factors consist of receptors such as platelet-derived growth factors (PDGF), epidermal growth factors (EGF), tumor necrosis factor-alpha (TNF-α), angiotensin II (AngII) and molecular advanced glycation end-products (RAGEs). All these receptors are protein tyrosine kinases (RTKs), which undergo auto-phosphorylation in response to ligand binding, which triggers the activation of NADPH-oxidase to elicit exogenous H_2_O_2_ which penetrates cells trough aquaporin. Protein-serine-threonine kinases and PTKs are under redox control of protein-tyrosine and protein-serine-threonine phosphatases (PTPs), which are inhibited by H_2_O_2_. Either activation of kinases or inhibition of phosphatases would shift the equilibrium toward phosphorylation. Cell exogenous generation of H_2_O_2_ by polyphenols inhibits PTPs, increasing phosphorylation through mostly activation by H_2_O_2_ of the PI3k/Akt/Nrf2 axis, increasing adaptation. The activation of protein kinases seems to be dependent on exogenous and endogenous H_2_O_2_ concentration and the duration of the stimuli which change the effects through activation of different transcription factors, leading to cell proliferation or adaptation (Eustress), or inflammation, apoptosis, necrosis and death (Distress).

Polyphenols ingestion at the right amount and time during the meal ameliorates the deleterious pathways in human organisms, delaying the development of many diseases by preventing generation of postprandial oxidative stress factors [132], and through the cardiovascular system induction in many organs, protects elements from oxidative stress. Polyphenols by both activities act synergistically for keeping the redox homeostasis in our organism, preventing diseases and balancing better human health.

## Figures and Tables

**Table 1:** Adaptation, protection and cell survival by preconditioning with H_2_O_2_ or polyphenols in cell-culture; in vitro systems.

Preconditioning with	Stress Compound	Model	Molecular Target	References
Plant/Compound				
H_2_O_2_ (10 µM)	H_2_O_2_ (6 mM)	COS cells	↑PI3K/Akt	[54]
H_2_O_2_ (100 µM, 10 min)	H_2_O_2_ (100 µM, 30 min)	Cardiomyocyte	↑PI3K/Akt/Nrf2	[55]
H_2_O_2_ (100 µM, 1.5 h)	H_2_O_2_ (100 µM, 30 h)	PC12 cells	↑PI3K/Akt/HO-1	[56]
Resveratrol (20 µM)	H_2_O_2_ (50 µM)	H9c2 cells	↑SirT1/SirT7, ↓caspase3	[59]
Resveratrol (100 µM)	H_2_O_2_ (1 mM)	C6 astrocytes	↑GSH/SOD/HO1, ↓ROS/iNOS	[60]
Resveratrol (100 µM)	Ethanol (0.78%)	Keratinocytes	↑GSH, ↓ROS	[57]
Baicalein (10 µM)	H_2_O_2_ (1 mM)	SH-SYSY cells	↑Nrf2/NQO1/SirT1,↓ necrosis	[58]
Hesperedin (1–50 µM)	H_2_O_2_ (0.4 mM)	PC12 cells	↑GSH-Px/cat,↓ LDH	[61]
Hydroxytyrosol (50 µM)	H_2_O_2_ (250 µM)	Endothelial cells	↑cat/AMPK/FOXO3,↓ ROS	[62]
Quercetin (25 µM)	H_2_O_2_ (60 µM)	Neurons cells	↑GSH/Nrf2/survival	[63]
Berry anthocy. (1 mg/mL)	H_2_O_2_ (500 µM)	ARPE-19	↑GSH-transferase/HO-1	[64]
Berry juice (10 µM gallate.eq)	H_2_O_2_ (750 µM)	N2a cells	↑GSH/SOD/MAPK/p-38	[65]

↑ = increase, ↓ = decrease. Gallic acid equivalent = gallate eq., Anthocyanins = anthocy.

**Table 2:** Adaptation, protection and animal survival by preconditioning with polyphenols via ingestion; in vivo.

Preconditioning with	Stress Compound	Model	Molecular Target	References
Plant/Compound				
Quercetin (40–80 mg/kg/d)	CCl_4_	Mice/liver	↓TLR2/MAPK/NFkB/ROS/MDA	[72]
Baicalein (80 mg/kg/×2/d)	CCl_4_	Mice/liver	↑TGF/EGF, ↓TNFα/IL6/ALT	[73]
Diecol (25 mg/kg/6/d)	CCl_4_	Mice/liver	↑SOD/CAT/GSH-Px, ↓MDA	[74]
Grape seed PP (150 mg/kg/d)	CCl_4_	Mice/liver	↑SOD/GSH-Px-Tx, ↓ALT/TNFα/IL6/MDA	[75]
Apple PP (200–800 mg/kg/d)	CCl_4_	Mice/liver	↑SOD/GSH, ↓ALT/MDA	[70]
Zingerone (40 mg/kg/d)	LPS	Mice/lung	↓TNFα/IL6/NFkB/MAPK	[76]
Curcumin (20 mg/kg/d)	LPS	Mice/liver	↓AST/TNFα/IL6/miRNA-155/PI3K/Akt	[77]
Berry PP (300 mg/kg/d)	LPS	Mice	↓Paw edema/TNFα/IL6/iNOS/NFkB, ↑Nrf2	[78]
Apigenin (20 mg/kg/d)	Arterial occlusion	Mice/brain	↓infarct area/microgalia	[79]
Anthocyanin (320 mg/d)	Dyslipidemia	Human	↓IL6/TNFα/MDA/8-iso-PGF_2α_/8-OHdG	[80]
Red Wine PP (150 mg/d)	Angiotensin II	Rat/endothelial	↓VEGF/MMP2/eNOS/ROS	[69]

↑ = increase, ↓ = decrease. TLR 2 = Toll–like receptor, NF-kB = (nuclear factor kappa B cells), MDA = malondialdehyde, GSH-Px = glutathione peroxidase, TGF = transforming grow factor, EGF = epidermal growth factor, TNFα = tumor necrosis factor α, IL6 = interleukin 6, ALT = alanine transaminase, AST—aspartate transaminase, miRNA-155 = microRNA-155, 8-iso-PGF_2α_ = 8-Iso-Prostaglandin F2α, 8-OHdG = 8-hydroxy-2′-deoxyguanosine, VEGF = vascular epidermal growth factor, MMP2 = matrix metalloproteinase-2, eNOS = endothelial nitric oxide synthase.
